# Deep Learning-assisted Diagnosis of Extrahepatic Common Bile Duct Obstruction Using MRCP Imaging and Clinical Parameters

**DOI:** 10.2174/0115734056363648241215145959

**Published:** 2025-01-17

**Authors:** Do Kieu Trang Thoi, Jung Hyun Lim, Jin-Seok Park, Suhyun Park

**Affiliations:** 1 Department of Electronic and Electrical Engineering, Ewha Womans University, Seoul 03760, South Korea; 2 Grado Department of Industrial and Systems Engineering, Virginia Tech, VA 24061, USA; 3 Division of Gastroenterology, Department of Internal Medicine, Inha University College of Medicine, Incheon 22332, South Korea

**Keywords:** Common bile duct, obstruction, magnetic resonance, cholangiopancreatography, deep learning approach, clinical parameter

## Abstract

**Background:**

Extrahepatic Common Bile Duct Obstruction (EHBDO) is a serious condition that requires accurate diagnosis for effective treatment. Magnetic Resonance Cholangiopancreatography (MRCP) is a widely used noninvasive imaging technique for visualizing bile ducts, but its interpretation can be complex.

**Objective:**

This study aimed to develop a deep learning-based classification model that integrates MRCP images and clinical parameters to assist radiologists in diagnosing EHBDO more accurately.

**Methods:**

A total of 465 patients with clinical data were included, of whom 143 also had MRCP images. Missing clinical values were addressed through data imputation. Object detection techniques were used to isolate the common bile duct region in the MRCP images. A multimodal deep learning fusion model was developed by combining the extracted imaging features with selected clinical parameters. To account for the varying significance of different features, a weighted loss function was applied. The performance of the fusion model was compared to that of single-modality approaches (using only MRCP images or clinical data), specifically the accuracy, sensitivity, specificity, and Area Under The Curve (AUC).

**Results:**

The performance of the proposed deep learning fusion model was superior to that of models using only MRCP images or clinical parameters. The fusion model achieved an accuracy of 89.8%, AUC of 90.4%, sensitivity of 81.8%, and specificity of 95.7% in diagnosing EHBDO. By integrating MRCP imaging data and clinical parameters, the proposed deep learning model significantly enhanced the accuracy of EHBDO diagnosis.

**Conclusion:**

This proposed multimodal approach outperformed traditional single-modality methods, presenting a valuable tool for improving the diagnostic accuracy of bile duct obstruction.

## 1 INTRODUCTION

Extrahepatic Common Bile Duct Obstruction (EHBDO) occurs when there is a blockage in the Common Bile Duct (CBD), impeding the flow of bile from the liver to the duodenum [1]. This condition can lead to severe complications, including jaundice, biliary infection, and pancreatitis, as the blockage often disrupts both bile and pancreatic enzyme drainage. Therefore, accurate and timely diagnosis of EHBDO is critical for effective treatment and favorable patient outcomes.

Although clinical parameters, including tumor markers, such as carbohydrate antigen 19-9 (CA19-9), have been found to aid in the diagnosis of bile duct obstruction, imaging techniques remain the cornerstone for confirming the diagnosis [1-3]. Magnetic Resonance Cholangiopancreatography (MRCP) is a noninvasive imaging modality that is widely used for visualizing the biliary and pancreatic ducts. Compared to endoscopic retrograde cholangiopancreatography (ERCP), MRCP provides detailed anatomical information without the risks associated with invasive procedures. However, the interpretation of MRCP images can be complex due to the anatomical variations and heterogeneity of bile duct obstructions, which may affect different regions of the biliary tree.

Despite the advantages of MRCP, its interpretation is largely manual, and diagnostic accuracy can vary between radiologists [4]. Studies have demonstrated that the sensitivity and specificity of MRCP for detecting biliary strictures and dilatations remain suboptimal, leaving ample room for improvement [5]. In recent years, advances in deep learning, particularly Convolutional Neural Networks (CNN), have shown promise in enhancing the diagnostic accuracy of various imaging modalities [6-10]. CNN-based models have been successfully applied to detect conditions, such as cholangiocarcinoma and common bile duct stones [7, 8]. However, currently there is a lack of deep learning models specifically designed to diagnose EHBDO using MRCP images.

To address this gap, this study explored a deep learning-based approach that integrates MRCP imaging data with clinical parameters to enhance the diagnosis of EHBDO. By employing a multimodal fusion model that combines features from both modalities, this study aimed to improve the accuracy of EHBDO diagnosis, surpassing the capabilities of single-modality approaches. The performance of the proposed model was compared with that of traditional machine learning and deep learning techniques based on either clinical parameters or MRCP images alone. The proposed approach not only provides a more comprehensive diagnostic tool but also represents a significant advancement in the field, with the potential to greatly impact clinical practice by assisting radiologists in making more accurate and efficient diagnoses of EHBDO.

## 2 MATERIALS AND METHODS

### 2.1 Patients and Dataset

The radiological and procedural data of patients who underwent MRCP for suspected pancreaticobiliary disease at a pancreaticobiliary center (Inha University Hospital, Incheon, Korea) between January, 2015 and December, 2021, were retrospectively analyzed. This study complied with the ethical guidelines of the 1975 Declaration of Helsinki [11], and the protocol was approved by the institutional review board (2021-08-015). The requirement for written informed consent was waived. Fig. (**1**) illustrates the patient data selection process. The patients were divided into two groups based on the diagnosis: normal (N) and obstruction (O) of the extrahepatic bile duct. The patient data included demographic information, blood test results, imaging reports (abdominal computed tomography [CT], MRCP, or ERCP), and surgical details. MRCP examinations were performed using the MRI scanners, OPTIMA MR450w 1.5T, SIGNA Architect 3.0T (GE Healthcare, Chicago, IL, USA) and Ingenia Elition 3.0T (Philips Healthcare, Boston, MA, USA). The MRCP images were analyzed by a biliary radiologist with >10 years of experience and two pancreaticobiliary specialists. Table **1** presents the information of patients in the two groups: normal (n = 72) and obstruction (n = 71). The radiologist interpreted the findings without knowledge of the original report.

The threshold for CBD dilation was defined as the widest diameter of the common bile duct being 11 mm or more in the vicinity of the portal vein. The final diagnoses in the obstruction group included cancer confirmed through histopathological examination, bile stones detected *via* ERCP, and benign biliary strictures (BBS) diagnosed based on histopathological findings and ERCP observations. Studies have suggested correlations between CBD obstruction and various blood indicators, including total bilirubin (TBIL), alkaline phosphatase (ALP), aspartate aminotransferase (AST), alanine aminotransferase (ALT), and tumor markers, such as CA19-9 [2, 12]. As mentioned in Table **1**, the clinical parameters between the obstruction and normal groups were compared using the t-test. Age, sex, ALP, and TBIL levels were significantly different between the two groups. Although CA19-9, AST, and ALT did not show statistically significant differences between the two groups, previous studies have established their correlations with a diagnosis of EHBDO [13, 14]. Thus, the clinical parameters of age, sex, and AST, ALT, TBIL, ALP, and CA19-9 levels were selected for inclusion in the deep learning-based model and were normalized within the range of 0–1. For cross-validation, the patients were divided into three folds, as shown in Fig. (**1**).

### 2.2 Data Imputation

As the diagnostic process varied among individuals, not all patients underwent the same set of tests, leading to incomplete parameters (% missing values [MV] in Table **1**). To address this, clinical imputation was performed using multiple imputations by chained equations [15] to estimate the MV in the clinical datasets. The multiple imputations by chained equations model [16] is an iterative approach that stops when the change in predictions is less than 10%. To enhance the robustness of the imputation process, clinical data from an additional 322 patients were utilized. These patients did not include the original cohort of 143 patients, who had both MRCP images and clinical data.

### 2.3 Pre-processing

Before classifying the EHBDO diagnosis, the CBD area in the MRCP image was identified using the YOLO model developed for object detection. The pretrained YOLO v7 [17] detection model was fine-tuned using the MRCP dataset with formatted annotations. For the CBD detection task, we specifically trained the YOLO v7 model to distinguish the CBD from the surrounding hepatic structures. The architecture of the model incorporates attention mechanisms that focus on regions of interest (ROI), effectively minimizing interference from neighboring structures and enhancing the detection accuracy. Bounding box annotation is a critical step in training the YOLO v7 model for precise localization of the CBD in MRCP images. Each patient’s MRCP image was carefully annotated with bounding boxes by an experienced biliary radiologist with over 10 years of expertise and two pancreaticobiliary specialists. This expert annotation ensured that the model concentrated specifically on the CBD region, thus reducing the risk of interference from adjacent anatomical structures. The CBD detection dataset comprised the medical records of 35 patients, with a corresponding number of medical images resized to 320 x 320 pixels. The training dataset consisted of 23 patient records with 197 medical images, whereas the validation dataset contained 10 patient records with 28 medical images. The CBD detection model was trained with a total of 300 epochs, using a batch size of 6 images per training iteration. The learning rate started at 0.01 and dynamically increased to a maximum of 0.1 during training using the one-cycle learning rate scheduler. The Adam optimizer was employed with a momentum of 0.937 and weight decay of 0.0005. Additionally, a warmup phase lasting 3 epochs was implemented, with a momentum of 0.8 and a specific learning rate of 0.1 for biases. Table **2** presents the performance of the CBD detection model. After CBD detection, the visible CBD data with the correct bounding box (c-1 in Table **2**) were further processed for the CBD obstruction classification task. Table **3** presents a summary of the dataset used for training and testing the CBD obstruction classification model. For each fold, the training set included 123 patients, while the test set included 20 patients for validation. It is important that the validation set contains the full clinical parameters. Table **4** details the augmentation methods used for training the CBD obstruction classification model.

### 2.4 Proposed Method

**Fig. (2):**
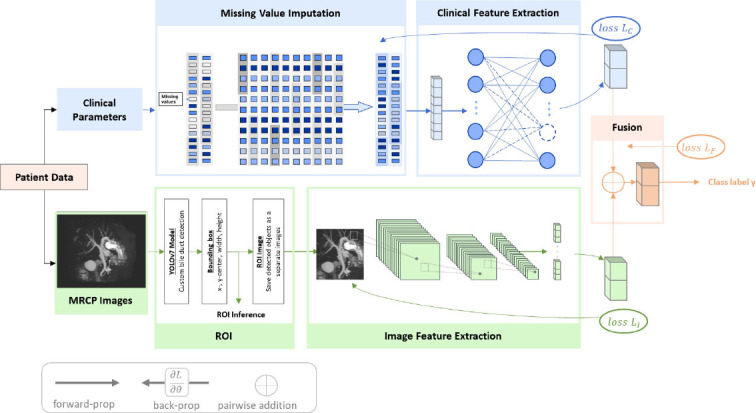
Proposed FM-TL model for diagnosing EHBDO. Features of the corresponding modality are extracted from clinical and image data. The features from multimodality data are fused to diagnose EHBDO.

A fusion model for diagnosing EHBDO was proposed by integrating features from both clinical parameters and MRCP images (Fig. **2**). To extract the relevant features from clinical parameters, a neural network model (clinical NN) with three layers, including a linear layer (hidden layers of sizes 100 and 50), ReLU activation, and dropout regularization, was used. For the extraction of image features, ResNet50 [18] was used on ROI in the CBD area, which was extracted during the pre-processing of MRCP images. As shown in Fig. (**2**), the extracted clinical features were fused with imaging features to create a comprehensive fusion model. To leverage the strengths of both modalities, an element-wise addition operation [19] was employed for the fusion of the complementary information derived from clinical parameters and images to enhance the discriminative power of the proposed fusion model.

To train the proposed fusion model, a multitask learning approach [20] was utilized, in which separate loss functions were defined for each modality, along with a joint loss function with the corresponding task weight for each task. The model was formulated with input samples *x* ϵ *X*, where *X* is the input space and labels (output) y from the label space *Y*. The input *x* is mapped by a mapping function *H_y_* (label predictor) to the label *y*, and the parameter representing this mapping is denoted as *θ_y_*, *i.e*., *y* = *H_y_*(*x*; *θ_y_*). The model aims to minimize the loss 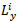 evaluated in *i*-th training data between the predicted probability 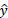 and target output y. A saddle point can be found as a stationary point of the following stochastic updates Eq (1):


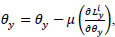


where *µ* is the learning rate. The weighted linear sum of the losses for each task (C: clinical parameters, *I*: images, and *F*: fusion) is defined as follows Eq (2):


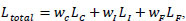


where, *w_c_* + *w_l_* + *w_F_* = 1 and *L_C_*, *L_I_*, and *L_^F^_* are the losses for the clinical parameters, images, and fusion, respectively. The cross-entropy loss was used as the loss function (*L_task_*) and calculated as follows Eq (3):


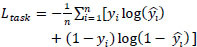


where *n* is the number of patients, *y* is the class label, and 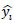 is the predicted probability. The weights *w_C_*, *w_I_*, *w_F_* determine the significance of the individual tasks, which were set as 0.25, 0.25, and 0.5, respectively. Since the proposed fusion model utilizes the total weighted loss, it is called FM-TL in this study.

To evaluate the performance of the proposed FM-TL model, we compared it with models that used only clinical parameters or images for classification. For the model based on clinical parameters alone, we utilized radiomic approaches from recent literature on bile duct obstruction, including a multilayer perceptron (MLP) classifier [21], LightGBM [3, 22], and Clinical NN [7], with the parameters optimized through grid search. For the image-only classification, models, such as EfficientNetB5 [23], InceptionNetV3 [24], and ResNet50, were used. These models, pretrained on ImageNet, were fine-tuned using the MRCP imaging data. The models were trained for 100 epochs with a batch size of 16 and an initial learning rate of 0.001, and the learning rate was decayed by a factor set of 0.1 in 50 and 75 epochs. We employed an SGD optimizer [25] with a Nestorov momentum of 0.9. All models were trained and tested using an Intel(R) Core (TM) i7-11700 CPU @ 2.50GHz processor, CUDA-enabled NVIDIA GeForce GTX 1660 Ti graphical processing unit, Python 3.8, and PyTorch 1.12. The performance of the models was evaluated in terms of accuracy, sensitivity, specificity, and receiver operating characteristic (ROC) curves. All numerical values are presented as means with confidence intervals and were analyzed using the t-test based on four iterations of three cross-validation folds. Gradient-weighted class activation mapping (Grad-CAM) [26] was utilized to visualize the regions that affected the model’s decision.

## 3 RESULTS

**Fig. (4):**
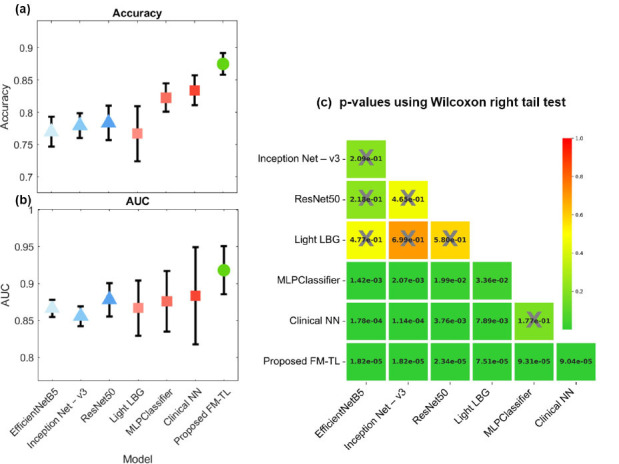
Performance evaluation using (**a**) accuracy and (**b**) AUC of the test dataset with the marker and vertical lines indicate the mean and confidence interval of relative improvements, respectively. The marker shapes of triangular, square, and circle represent the model for image data, clinical parameters, and the proposed FM-TL model, respectively. (**c**) Statistical significance (*p*<0.05 rejects the null hypothesis: same performance) was calculated by the Wilcoxon signed-rank test.

The proposed FM-TL model combining clinical parameters and MRCP imaging data showed the best performance in classifying EHBDO diagnoses among all models, as presented in Table **5** and Fig. (**3**). Fig. (**3a**) shows the ROC curves of the ResNet50 model based on MRCP images alone, the NN model based on clinical parameters alone, and the proposed FM-TL model. Fig. (**3b**) shows the mean ROC curves with a standard deviation range of three folds and ROC curves for the individual folds. The differences between the models are highlighted in Fig. (**4**), including the accuracy (Fig. **4a**), the area under the curve (AUC, Fig. **4b**), and p-values based on the Wilcoxon right-tail test (Fig. **4c**). The accuracy of the proposed FM-TL model (the least p-value) was superior to that of the models based solely on clinical parameters or MRCP images.

Fig. (**5**) shows the representative MRCP images of patients with normal CBD, BBS, bile stones, and cancer. Fig. (**5a**) shows the MRCP images, Fig. (**5b**) demonstrates the detected CBD area after data pre-processing, and Fig. (**5c**) shows the Grad-CAM for the successful prediction of the position and presence of EHBDO using the proposed FM-TL model, where the probabilities of prediction of normal CBD, BBS, bile stone, and cancer were 0.084, 0.868, 0.906, and 0.999, respectively. The results confirmed that the proposed model is accurate and reliable for CBD detection and obstruction classification.

**Fig. (6):**
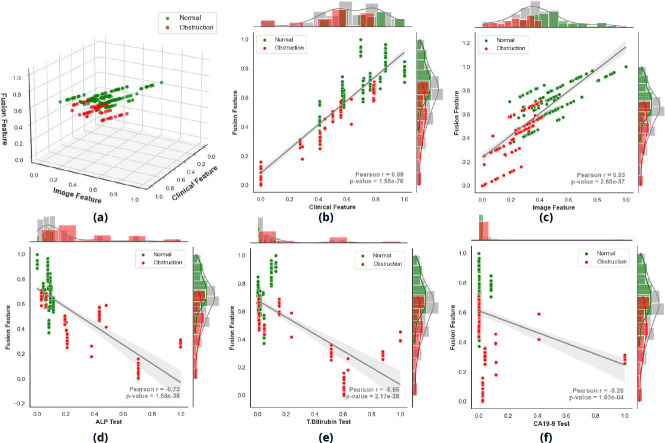
Scatter plots of correlations (**a**-**c**) between image, clinical, and fusion features and (**d**-**f**) between three traditional clinical parameters, including ALP, TBIL, and CA19-9, and fusion features.

The correlations of the features extracted from clinical parameters and imaging data with the fusion features are depicted in Fig. (**6**). The results indicate that both the images and clinical features have a strong correlation (above 0.8) with the fusion features (Fig. **6a**-**c**). Among the clinical features, ALP showed the highest correlation, followed by TBIL. In contrast, CA19-9 showed a weaker correlation, potentially because of its inconsistent performance in distinguishing between normal and obstruction cases. These findings suggest that ALP is the most reliable clinical predictor of EHBDO.

## 4 DISCUSSION

In this study, we established a deep learning model, FM-TL, by integrating MRCP imaging and clinical parameters for a more comprehensive diagnosis of EHBDO. By combining a detailed visualization of biliary structures in MRCP images with the relevant clinical data, the model demonstrated superior diagnostic performance over traditional diagnostic methods. This approach can help to improve the diagnostic accuracy of EHBDO.

MRCP image-based diagnostics are often associated with variability due to factors, such as acquisition artifacts, image orientation, and intensity inconsistencies due to anatomical variations [27]. The FM-TL model effectively managed these challenges by using image augmentation techniques, particularly for the detection of CBD. This model can successfully identify the CBD, demonstrating an accuracy of 81.39% despite potential interference from surrounding structures in the hepatobiliary and pancreatic systems. However, some CBD geometries were still missing in the analyzed dataset, and future studies should expand the training dataset to include a broader range of anatomical variations, which can enhance the accuracy and robustness of the model in clinical applications.

One major limitation of this study is its relatively small sample size. Bile duct obstruction often results from malignancies, such as pancreatic cancer, cholangiocarcinoma, and metastatic tumors. However, the incidence of these cancers is low, making it challenging to collect a sufficient number of relevant MRCP images. Despite the limited sample size, our results demonstrated the feasibility of using this approach in clinical practice. Nevertheless, a larger dataset with an external validation group is required for further validation of this approach. Another limitation was the retrospective nature of this study, which may have led to bias and missing values of the clinical parameters. For example, there was a higher proportion of men with bile duct obstruction, and individuals in the normal group were generally older than those in the obstruction group. To address the missing values, clinical imputation was performed. Additionally, several clinical parameters were excluded due to the high rate of missing values or statistical insignificance. Tumor markers, such as CA19-9 and carcinoembryonic antigen (CEA), are considered important diagnostic factors. However, CEA was excluded due to a high rate of missing data (up to 90% missing values). The selection of clinical parameters was based on both their availability and relevance to bile duct obstruction in order to balance data completeness with clinical utility. These biases and missing data may impact the generalizability of the results, and future studies should focus on improving the data collection methods to reduce these limitations.

EHBDO can be caused by both malignant and benign tumors, making the classification of malignancy a critical aspect of EHBDO diagnosis. In this study, 49.3% and 50.7% of patients in the obstruction group had malignant and benign tumors, respectively. When tested on MRCP images by a patient-based analysis using majority voting, the model achieved accuracy, sensitivity, and specificity of 80.0%, 70.0%, and 90.0%, respectively. While this result highlights the model's effectiveness in detecting EHBDO and distinguishing between benign and malignant tumors, the limited dataset constrained its ability to differentiate between specific subtypes of malignant and benign obstructions. Future research should prioritize collecting a larger and more balanced dataset to address this limitation and enhance the model's diagnostic capabilities.

Despite its strong performance, the sensitivity of the FM-TL was affected by the variability in MRCP images, which led to lower sensitivity scores compared to those of models that used only clinical parameters. Clinical data, while more objective, are susceptible to errors when other structures surrounding the hepatobiliary and pancreatic systems are obstructed. However, the key strength of the FM-TL model lies in its ability to reduce false positives, resulting in significantly better specificity compared to that of single-modality approaches. Further refinement of the model, particularly the management of image variability, will be crucial for optimizing its overall diagnostic performance.

Another challenge is differentiating EHBDO caused by compression from adjacent organs, such as the gallbladder, blood vessels, or pancreas, based solely on MRCP images. In clinical practice, additional imaging modalities, such as CT or ERCP, are often used to complement the MRCP findings. MRCP is a completely non-invasive imaging modality, making it an attractive initial diagnostic choice as it carries no procedural risks, unlike endoscopic ultrasound (EUS), which is minimally invasive and requires sedation and endoscopic insertion. MRCP also allows for a broader anatomical view of the hepatobiliary and pancreatic systems in a single session, which can be advantageous for assessing the extent of biliary obstruction and related anatomical variations. Furthermore, MRCP can be particularly useful for patients with high procedural risks or those who may not tolerate invasive procedures well, such as older patients or those with multiple comorbidities. Given these benefits, MRCP serves as a safer, first-line diagnostic tool for EHBDO. However, EUS remains valuable for cases where further diagnostic clarity is needed or when tissue sampling is required, and it may be considered in future studies as a complementary tool in conjunction with MRCP. Future studies should consider incorporating supplementary modalities, such as EUS, into the model to improve the diagnostic accuracy in complex cases, such as in those with intrahepatic bile duct cancer, where neighboring structures can interfere with the diagnostic prediction.

## CONCLUSION

This study introduces a deep learning fusion model that integrates MRCP imaging and clinical parameters for improving the diagnosis of EHBDO. Despite the limitations in data availability, this model represents a significant advancement in diagnosing EHBDO, as it outperforms single-modality approaches in detecting CBD obstruction. Moving forward, the expansion of the dataset and incorporation of additional imaging techniques will further enhance the ability of the model to differentiate between malignant and benign causes of EHBDO, thus addressing the challenges identified in this study and improving diagnostic precision.

## Figures and Tables

**Fig. (1):**
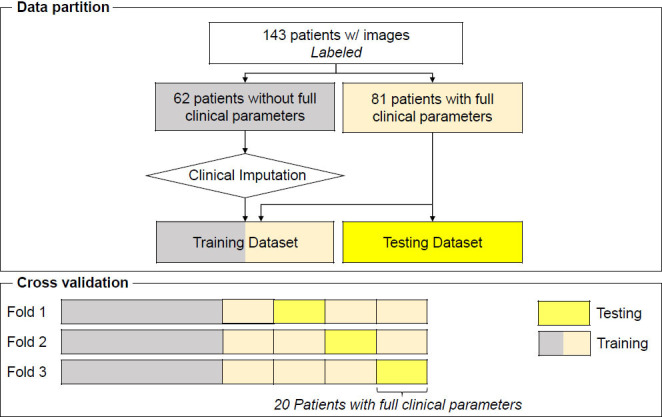
Flowchart of patient data selection for training and testing data sets.

**Fig. (3):**
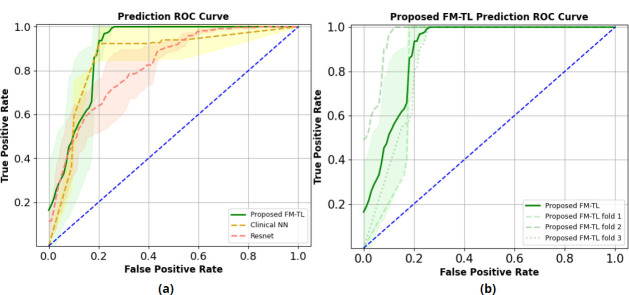
(**a**) Prediction ROC curves (Mean ROCs and CIs) of ResNet50 with MRCP images, NN model for clinical parameters, and proposed FM-TL, (**b**) prediction ROC curves of proposed FM-TL in three test folds.

**Fig. (5):**
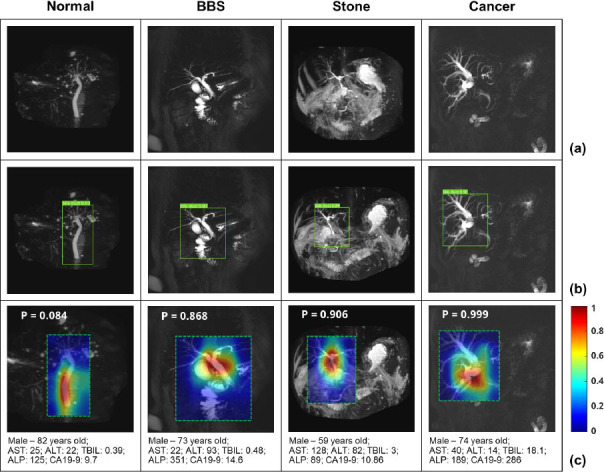
Examples of (**a**) MRCP images, (**b**) detected CBD area, and (**c**) the Grad-CAM of normal, BBS, stone, and cancer cases, respectively.

**Table 1:** Characteristics of patients in the obstruction and normal groups.

-	**Total**		**Obstruction**		**Normal**		-
**Patients**	n = 143		n = 71		n = 72		-
**Total images**	2650		1343		1307		-
**Parameters**	**% MV/ Value**		**% MV/ Value**		**% MV/ Value**		**p-value**
**Age (years) *[a]***	0	66.69 ± 14.43	0	63.1 ± 16.52	0	70.32 ± 10.91	<0.001
**Sex *[b]***	-	-	-	-	-	-	<0.014
Male	0	74	0	44	0	30	-
Female	0	69	0	27	0	42	-
**ALP (U/L) *[c]***	11	133 (81.5–275)	1	220.5 (134.5–382.5)	21	85 (64–125)	<0.001
**TBIL (mg/dL) *[c]***	25	1.21 (0.57–3.84)	13	2.84 (0.97–7.88)	38	0.73 (0.44–1.38)	<0.001
**ALT (IU/L) *[c]***	10	59.5 (24–155.75)	0	103 (37.5–190.5)	21	27 (17–77)	0.124
**CA19-9 (U/mL) *[c]***	24	17.6 (9–113)	15	71.89 (17.45–215.72)	32	9.9 (5.8–15)	0.425
**AST (IU/L) *[c]***	10	55 (24–118)	0	82 (43–132.5)	19	30 (20.25–67.5)	0.664

**Note: **(a) means ± standard deviation. (b) number of patients, with the percentage in parentheses. (c) median, with interquartile range in parentheses. TBIL, total bilirubin; ALP, alkaline phosphatase; AST, aspartate aminotransferase; ALT, alanine aminotransferase; CA19-9, carbohydrate antigen 19-9; MV, missing value.

**Table 2:** Visible CBD data selection from the CBD detection model.

-		**Total Images**	
		Images	%
**(a) Original MRCP images**		2650	-
**(b) Invisible CBD**		845	
**(c) Visible CBD – CBD detection model**		1805	100.00
**(c-1)**	Correct bounding box	1469	81.39
**(c-2)**	No bounding box	216	11.97
**(c-3)**	Incorrect bounding box	120	6.65

**Abbreviations: **CBD, common bile duct; MRCP, magnetic resonance cholangiopancreatography.

**Table 3:** Dataset used for training and testing of the CBD obstruction classification model.

**Data**		**Obstruction**		**Normal**		**Total**	
		Patients	Images	Patients	Images	Patients	Images
**Train**	Fold 1	61	561	62	685	123	1246
	Fold 2	61	557	62	712	123	1269
	Fold 3	61	575	62	709	123	1284
**Test**	Fold 1	10	94	10	129	20	223
	Fold 2	10	98	10	102	20	200
	Fold 3	10	80	10	105	20	185
**Total**		71	655	72	814	143	1469

**Abbreviation: **CBD, common bile duct.

**Table 4:** Image augmentation methods used for the CBD obstruction classification model.

**Transformation**		**Range**
**Resize image**		-
-	Resnet, VGG, Efficient Net	Height = Width = 224
-	Inception Net	Height = Width = 299
-	Mean = 0.257, STD = 0.109	-
**Normalization**		Probability = 0.5
**Random Horizontal Flipping**		Kernel size = (5, 9), Sigma = (0.1, 5)
**Gaussian Blur**		Sharpness factor = 2, Probability = 0.5

**Abbreviation: **CBD, common bile duct.

**Table 5:** Performance of classification models based on clinical parameters alone, imaging data alone, and the proposed FM-TL model.

**Model**	**Clinical Parameters**	**MRCP Images**	**Accuracy**	**AUC**	**Sensitivity**	**Specificity**
**Light GBM**	**√**	-	0.767 ± 0.043	0.867 ± 0.037	0.895 ± 0.070	0.664 ± 0.043
**MLP Classifier**	**√**	-	0.823 ± 0.022	0.876 ± 0.041	0.873 ± 0.063	0.776 ± 0.071
**Clinical NN**	**√**	-	0.833 ± 0.023	0.883 ± 0.066	0.870 ± 0.097	0.799 ± 0.081
**EfficientNetB5**	-	**√**	0.770 ± 0.023	0.866 ± 0.012	0.837 ± 0.026	0.713 ± 0.055
**InceptionNetv3**	-	**√**	0.779 ± 0.019	0.856 ± 0.013	0.791 ± 0.033	0.768 ± 0.040
**ResNet50**	-	**√**	0.783 ± 0.027	0.878 ± 0.022	0.846 ± 0.020	0.729 ± 0.060
**Proposed FM-TL**	**√**	**√**	**0.874 ± 0.017**	**0.918 ± 0.033**	**0.829 ± 0.050**	**0.907 ± 0.039**

**Abbreviations: **MLP, multilayer perceptron; MRCP, magnetic resonance cholangiopancreatography; AUC, area under the curve.

## Data Availability

The data and supportive information are available within the article.

## LIST OF ABBREVIATIONS

CBD: Common Bile Duct
AUC: Area Under The Curve
MRCP: Magnetic Resonance Cholangiopancreatography
EHBDO: Extrahepatic Common Bile Duct Obstruction

## STANDARDS OF REPORTING

STROBE guidelines were followed.
